# Comparison of Diagnostic Ability Between Wide-Field Swept-Source Optical Coherence Tomography Imaging Maps and Heidelberg Retina Tomograph 3 Optic Nerve Head Assessment to Discriminate Glaucomatous and Non-glaucomatous Eyes

**DOI:** 10.7759/cureus.28188

**Published:** 2022-08-19

**Authors:** Dimitrios Kourkoutas, George Triantafyllopoulos, Iordanis Georgiou, Aristotelis Karamaounas, Nikolaos Karamaounas, Konstadinos Sotiropulos, Dimitrios Kapralos

**Affiliations:** 1 Ophthalmology, 401 Army General Hospital, Athens, GRC; 2 Optometry, 401 Army General Hospital, Athens, GRC; 3 Ophthalmology, “G. Gennimatas” General Hospital of Athens, Athens, GRC; 4 Ophthalmology, Euroclinic, Athens, GRC; 5 Ophthalmology, 417 Army Share Fund Hospital, Athens, GRC

**Keywords:** diagnostic ability, glaucoma, heidelberg retina tomograph, wide-field optical coherence tomography, swept-source optical coherence tomography

## Abstract

Background

In this study, we aimed to determine the diagnostic performance of optic nerve head (ONH), macular, and circumpapillary retinal nerve fiber layer (cpRNFL) thickness measurements of wide-field maps (12 × 9 mm) using swept-source optical coherence tomography (SS-OCT) compared to measurements of the ONH and RNFL parameters measured by Heidelberg Retina Tomograph (HRT3).

Methodology

This case-control study included 39 eyes of 39 glaucoma patients and 36 eyes of 36 normal subjects (control group). All participants underwent standard automated perimetry (SAP) as well as structural measurements by SS-OCT (DRI-OCT, Triton; Topcon Inc., Tokyo, Japan) and HRT3 (Heidelberg Engineering, Heidelberg, Germany). The abilities of the continuous parameters to discriminate between glaucoma and control groups were assessed using areas under the receiver operating characteristic curves (AUCs). To assess the glaucoma diagnostic abilities of each of the categorical variables, sensitivity, specificity, positive predictive value, and negative predictive value were tested.

Results

The highest sensitivities were achieved by the DRI-OCT categorical parameters of Superpixel-200 map and cpRNFL (12 sectors) thickness analysis. The best performing HRT3 continuous parameter was rim volume (AUC = 0.829, 95% confidence interval (CI) = 0.735-0.922), and the best continuous parameter for DRI-OCT wide-field was vertical cdr (AUC = 0.883, 95% CI = 0.805-0.951), followed by total cpRNFL thickness (AUC = 0.862, 95% CI = 0.774-0.951). AUCs for disc area, rim area, linear cdr, and RNFL thickness were not significantly different between the two technologies. Using either the most or the least specific criteria, SuperPixel-200 map always showed the highest sensitivity among the categorical parameters of both technologies (82.1% and 89.7%, respectively). The highest sensitivity among HRT3 classification parameters was shown by MRA and GPS classification algorithms.

Conclusions

Both wide-field DRI-OCT maps and HRT3 showed good diagnostic performance in discriminating glaucoma. Although DRI-OCT thickness values and normative diagnostic classification showed the best performance, more studies are required to determine the clinical role of wide-field DRI-OCT scan in glaucoma diagnosis.

## Introduction

Glaucomatous optic neuropathy involves characteristic optic disc as well as retinal nerve fiber layer (RNFL) structural damage and related functional defects [1]. Even though structural and functional tests are not always correlated, assessment of both visual function and structure is recommended for diagnosis and disease monitoring [1,2]. Widely utilized methods in objectively assessing glaucomatous structural changes include the long-accepted Heidelberg Retinal Tomograph 3 (HRT3, Heidelberg Engineering GmbH, Heidelberg, Germany) and the continuously evolving technology of optical coherence tomography (OCT). These instruments provide a quantitative evaluation of optic nerve head (ONH), RNFL, and macula anatomic structural parameters; compare these measurements with normative data; and allow the documentation of data. Therefore, these instruments can provide a more objective method of diagnosing and monitoring glaucoma [3].

The HRT3 is a confocal scanning laser tomography (CSLO) device that uses a diode laser (670 nm) to scan the retinal surface at multiple consecutive parallel focal planes and produces repeatable and reproducible three-dimensional (3D) topographical images of the ONH and peripapillary RNFL [4]. After image acquisition, the margins of the ONH need to be outlined by a manually drawn contour line to calculate ONH stereometric parameters. HRT3 also provides two different algorithms for ONH anatomy classification: the Moorfields regression analysis (MRA) that requires a contour line to be placed [5], and the newer contour-line independent Glaucoma Probability Score (GPS) [6]. The quantitative and objective measures of these structures are consequently classified as within normal limits (WNL), borderline, or outside normal limits (ONL) by automatic comparison with an ethnic-selectable normative database of eyes [7]. Several researchers have shown that HRT3 is useful in diagnosing [8,9] and monitoring glaucoma progression [10-13].

Deep range imaging OCT (DRI-OCT, Triton, Topcon, Tokyo, Japan) is a recently introduced swept-source OCT (SS-OCT) that uses a center wavelength of 1,050 nm and a bandwidth of approximately 100 nm compared to the fixed 850 nm wavelength of spectral-domain OCT (SD-OCT) [14,15]. The instrument achieves a high scan speed (100,000 A-scans/second) that allows for the acquisition of high-quality wide-field images containing both the ONH and the macula in a 12 mm × 9 mm single scan. SS-OCT, similar to SD-OCT, also provides separate standard macula and optic disc scan modes. Both thickness measurement values and normative comparisons are provided for all SS-OCT measurements.

Recently, several investigators have reported similar glaucoma diagnostic abilities of SS-OCT and SD-OCT standard macula and disc scans [16] as well as between wide-field SS-OCT and standard SD-OCT macula or disc scans [17-20], while another study showed good repeatability of SS-OCT [19]. In addition, the wide-field scan showed a similar glaucoma detection ability as standard macula/disc scans by SS-OCT [21].

Our study aimed to assess the ability of the 3D Wide Glaucoma Report, which is generated using wide-field DRI-OCT scans, to distinguish glaucoma from healthy eyes. The diagnostic ability of the wide-field-based automatic classification was compared with that of the six main HRT3 stereometric parameters as well as the GPS and MRA analyses in the discrimination of glaucomatous and healthy eyes. Glaucoma diagnostic abilities of wide-field DRI-OCT and HRT3 thickness measurement values were also compared.

## Materials and methods

Study participants

This was a case-control study that included 36 eyes of 36 normal subjects (controls) and 39 eyes of 39 glaucoma patients who visited the Glaucoma Clinic of 401 Army General Hospital of Athens between August 2019 and July 2020. The study protocol complied with the Declaration of Helsinki and was approved by the Institutional Review Board of 401 Army General Hospital of Athens (approval number: 3/2019). Informed consent was obtained from all participants.

Each participant underwent comprehensive ophthalmic tests, including medical history review, best-corrected visual acuity (BCVA) by Snellen digital chart, slit-lamp biomicroscopy, intraocular pressure (IOP) measurement by Goldman applanation tonometry, gonioscopy, dilated fundus examination, and fundus stereophotographs. Standard automated perimetry (SAP) using SITA 24-2 strategy (Humphrey Field Analyzer, model 740 ii; Carl Zeiss Meditec, Dublin, CA, USA), wide-field SS-OCT (DRI-OCT Triton; Topcon, Tokyo, Japan) imaging, and CSLO imaging using the HRT3 (Heidelberg Engineering, GmbH, Heidelberg, Germany) were also performed on the same day as the ophthalmic examination.

Inclusion criteria included a BCVA of 20/40 or better, spherical equivalent refractive errors between +6.0 and -6.0 D, cylinder correction of <3.0 D, and an open angle of the anterior chamber. All DRI-OCT images had a minimum image quality value (IQV) of 60. The recommended IQV is 40 according to the manual of the device (median value 58) [22]. All HRT3 images had an SD of >50 µm, even image exposure, and good centering. Exclusion criteria included any retinal pathology as well as systemic disease or systemic medication known to affect RNFL thickness or visual fields (VF). We also excluded eyes with a history of previous ocular surgery as well as any other macular disease that could interfere with segmentation of retinal layers by OCT. If both eyes met all inclusion criteria, one eye was randomly selected.

Eyes were classified either as healthy (control group) or glaucomatous (glaucoma group) based on the VF test results and the ONH appearance [23]. VF tests and fundus stereophotographs of all participants were analyzed by a glaucoma specialist (DK) masked from any other clinical data.

Control group consisted of subjects who satisfied all the following criteria in both eyes: (1) no previous intraocular surgery, (2) IOPof ≤22 mmHg, (3) clinically normal disc appearance, (4) a normal VF result defined as a mean deviation (MD) and pattern standard deviation (PSD) within 95% confidence limits (CIs) and a glaucoma hemifield test (GHT) result WNL, and (5) no other signiﬁcant ophthalmic ﬁndings.

Eyes were categorized as glaucomatous when there was glaucomatous structural damage (neuroretinal rim notching or thinning, or presence of an RNFL defect) and associated repeatable (≥2 consecutive) VF defects. Glaucomatous VF defect was defined [24] by a GHT ONL on at least two VFs; or a cluster of three or more non-edge points in a location typical for glaucoma, all of which are depressed on the pattern deviation plot at p < 5% level and one of which is depressed at a p < 1% level; or a PSD with p < 5% level.

HRT3

The most recent version of HRT technology, the HRT3 (Heidelberg Engineering GmbH, Heidelberg, Germany) was used to perform CSLO imaging. Briefly, a 3D topographic image consisting of 384 × 384 × 16 up to 384 × 384 × 64 pixels was constructed from multiple focal planes axially along the ONH. A mean topography image was created by averaging and aligning three consecutive scans of the ONH and RNFL. An experienced glaucoma specialist (DK) reviewed all images for the imaging score and the overall quality score and outlined the ONH margin on the mean topographic image. After the contour line was delineated, all the ONH stereometric measurements were automatically calculated.

**Figure 1:**
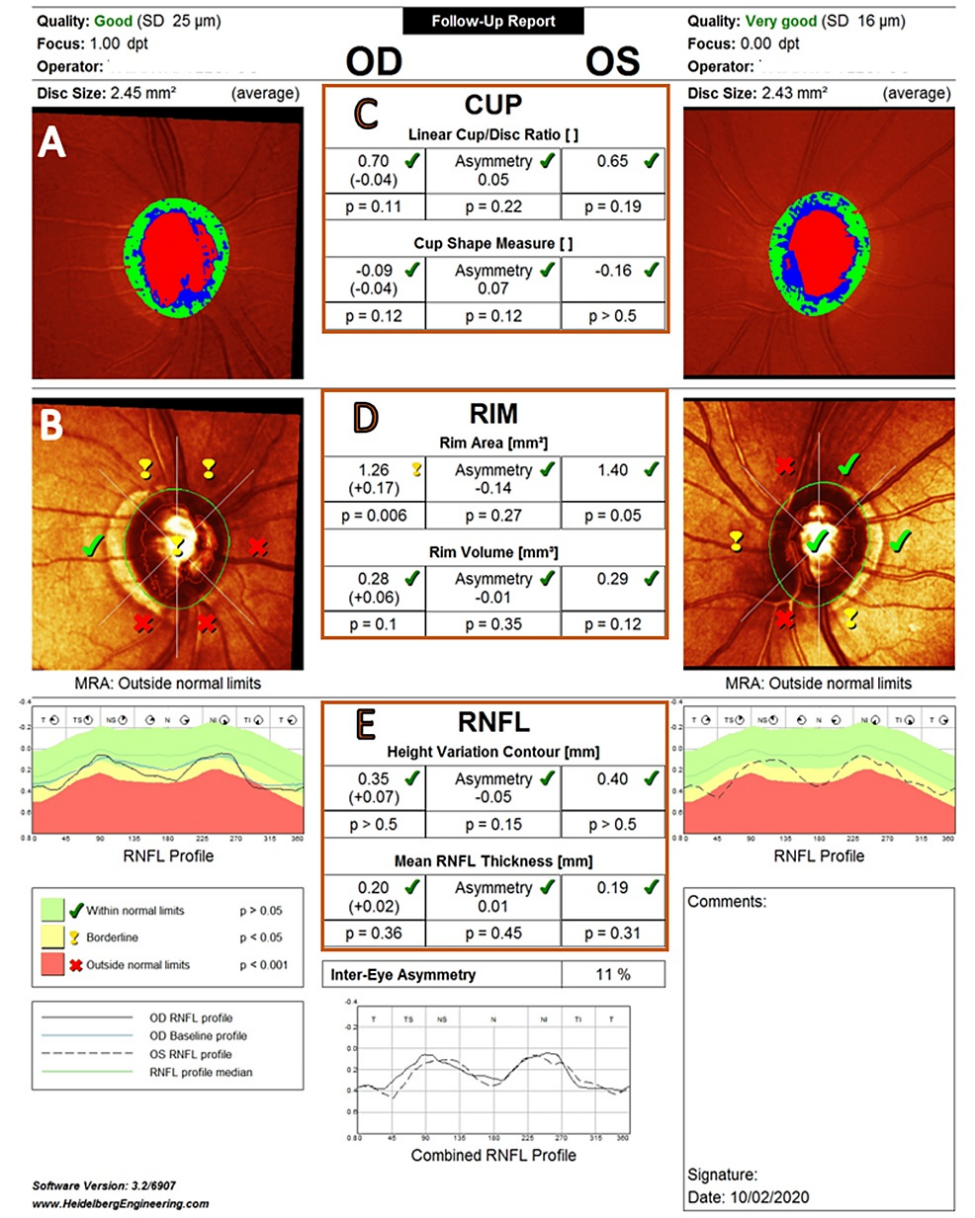
HRT3 OU report. A 63-year-old male with primary open-angle glaucoma in both eyes. (A) Topography image of the ONH. (B) MRA global and MRA classification. (C) cup, (D) rim, and (E) RNFL numeric stereometric measurement values and their categorical classification* (six main stereometric ONH parameters). *For categorical classification, each parameter is designated as WNL, borderline, or ONL after comparing with the normative database. HRT3 = Heidelberg Retinal Tomograph 3; ONH = optic nerve head; MRA = Moorfields regression analysis; RNFL = retinal nerve fiber layer; WNL = within normal limits; ONL = outside normal limits

The HRT3 OU report provides arithmetic (continuous) measurements of the six main ONH stereometric parameters (linear c/d ratio, cup shape measure (CSM), rim area, rim volume, height variation contour, mean RNFL thickness) (Figure 1).

In addition to stereometric parameters, the HRT3 offers two different automatic classification algorithms of the ONH morphology, the MRA which requires the placement of the contour line and the contour line-independent GPS. Stereometric parameters and classification algorithms of the ONH were compared to the values of the normative dataset and between the eyes and were consequently designated as WNL, borderline, or ONL. The HRT3 normative database contains 733 healthy Caucasian eyes and 215 healthy African American eyes [7]. Both MRA and GPS provide a global (MRA global - GPS global) as well as a final result (MRA classification - GPS classification). Normal classification requires that all sectors as well as the global result to be WNL. The presence of either at least one borderline sector or a borderline global result results in a borderline classification, while an ONL classification requires the presence of at least one ONL sector or an ONL global result.

DRI-OCT

DRI-OCT (DRI-OCT, Triton, Topcon, Tokyo, Japan) is a recently introduced SS-OCT with a longer wavelength of 1,050 nm and a sweeping range of approximately 100 nm compared to the fixed 850 nm wavelength typical of SD-OCT [14,15] that enables a deeper imaging range and better tissue penetration. The instrument has a scanning speed of 100,000 A-scans per second compared to the typical 40,000 A-scans per second scanning rate of SD-OCT devices. Consequently, the faster scan speed of DRI-OCT significantly reduces motion artifacts and facilitates the acquisition of a high-quality wide-angle scan image containing more image data in a single scan. The protocol 3D wide scan covers a large area just beyond ONH and arcades (12 mm × 9 mm), which comprises 256 B-scans, each comprising 512 A-scans (resolution 512 × 256). Consequently, this protocol can cover both the macula and the ONH in 1.3 seconds with an axial resolution of 8 μm in the tissue [25].

**Figure 2:**
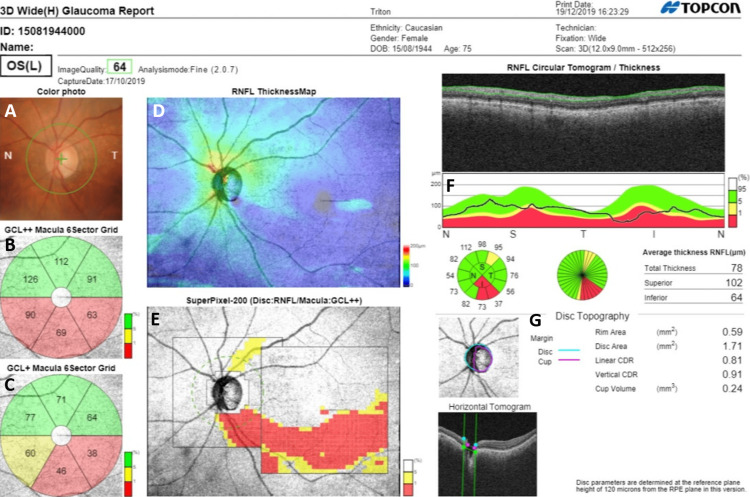
DRI-OCT Triton: 3D wide(H) glaucoma report. A 75-year-old female with primary open-angle glaucoma in her left eye. (A) Conventional color photography of the ONH. (B, C) macular GC analysis*. (D) Color-coded RNFL thickness map that corresponds to numeric RNFL thickness measurements. (E) SuperPixel-200 map. The uncolored pixels indicate the normal range, whereas the yellow- and red-colored pixels indicate abnormality at P = 1-5% and P < 1% of the normal level, respectively. (F) cpRNFL analysis*. (G) Numeric measurements of five ONH parameters. *Green-colored sectors indicate WNL thickness (measurement value is above 5% of the normative database values), whereas yellow-colored sectors indicate borderline thickness (measurement value is between 1-5% of the normative database values), and red-colored sectors indicate ONL thickness (measurement value is <1% of the normative database values). DRI-OCT = deep range imaging optical coherence tomography; ONH = optic nerve head; GC = ganglion cell; RNFL = retinal nerve fiber layer; cpRNFL = circumpapillary retinal nerve fiber layer; WNL = within normal limits; ONL = outside normal limits

Automated segmentation of the retinal layers by the built-in software is a crucial tool for quantitative and qualitative tissue evaluation. The thickness measurements of retinal layers (circumpapillary (cp)RNFL, RNFL, and macular layers) are compared to a normative data and a single-page wide-field report (3D wide glaucoma report) (Figure 2) is generated that provides the following information: (1) optic disc color photo. (2) cpRNFL is measured on a 3.4 mm diameter circle automatically placed around the ONH, and measurement data is given in the form of a TSNIT graph, a peripapillary RNFL 4 quadrant map, a peripapillary RNFL 12 clock hour map, and a peripapillary RNFL 36 direction map. Each sector of the peripapillary RNFL map is color-coded following comparison with the normative database (categorical variables). (3) Numeric measurements (continuous variables) of five ONH parameters (rim area, disc area, cup volume, vertical, and horizontal c/d ratio) are also provided without normative comparison. (4) The macular layer analysis is performed by applying a circle 6 mm in diameter automatically centered on the fovea. The thickness of the following layers is calculated: (a) mGCIPL (GCL+): macular GC layer plus the inner plexiform layer (IPL) and (b) mGCC (GCL++): GCL+ plus RNFL. Mean thicknesses are measured for each of the six sectors of a macular grid. For each macular sector, both mean thickness measurement values and normative data color-coded analysis (categorical variables) are displayed. (5) The wide-field RNFL thickness map provides color scales that correspond to RNFL thickness measurement values within the 12 mm × 9 mm wide-field area. (6) The SuperPixel-200 map (RNFL deviation map) consists of 26 × 26 grids within a 5.2 × 5.2 mm^2^ peripapillary area (RNFL) and 30 × 30 grids within a 6.0 × 6.0 mm^2^ macular area (GC layer + IPL + RNFL). This wide-field RNFL deviation map provides a significance map by comparing the patient’s RNFL/RNFL + IPL + GC data with a built-in normative database. In the normative data color-coded maps, the green sectors indicate normal thickness measurement ranges, whereas yellow indicates borderline values of 1-5%, and red-colored sectors indicate values outside the normal range (<1% below the normal level). The DRI-OCT Triton normative database includes 410 healthy eyes from a wide range of ethnicities including Asian, Caucasian, Hispanic/Latino, African American, and others [26].

Definitions of glaucomatous structural change on wide-field DRI-OCTscan

Glaucomatous structural change on the categorical parameters of cpRNFL (four sectors), cpRNFL (12 sectors), mGCIPL (six sectors), and mGCC (six sectors) was defined as follows: (A) a WNL classification required all sectors to be WNL (green-colored), (B) a borderline classification occurred when at least one of the sectors was borderline (yellow-colored), and (C) an ONL classification occurred when at least one sector was ONL (red-colored).

Glaucomatous structural change on the SuperPixel-200 map was decided when 20 or more contiguous pixels (>20 pixels) with statistically significant change (yellow/red pixels) were identified within the scanned peripapillary and/or macular area.

Statistical analysis

Student’s t test was used to compare continuous thickness parameters between normal subjects and glaucoma patients, and the chi-square test was used for categorical parameters.

The abilities of the continuous parameters to discriminate between glaucoma and control groups were assessed using areas under the receiver operating characteristic curves (AUCs). We also calculated sensitivities at fixed specificities (80% and 90%) for each of these parameters. AUCs of the continuous parameters that are common to both technologies underwent paired comparisons using the method described by DeLong et al. [27]. To assess the abilities of each of the categorical variables based on a comparison of measurements with the built-in normative database to distinguish subjects with glaucoma from healthy controls, sensitivity and specificity, positive predictive value (PPV), and negative predictive value (NPV) were tested. McNemar test was used to compare sensitivity and specificity.

For categorical parameters, borderline classification could be considered either as WNL and then the classification was assessed as “most specific criteria” (resulting in increased specificity/decreased sensitivity), or borderline classification could be considered as ONL, and then the classification was assessed as “least specific criteria” (resulting in a decreased specificity/increased sensitivity). Statistical analyses were conducted using SPSS version 26 (IBM Corp., Armonk, NY, USA). For all tests, the level of statistical significance was set at P < 0.05.

## Results

Initially, 96 consecutive Caucasian patients were recruited in this case-control study. All participants visited the Glaucoma Clinic of 401 Army General Hospital in Athens from May 2019 through May 2020. After applying the inclusion criteria, 10 candidates were excluded for unreliable VF results, seven candidates for poor OCT image quality, and four candidates for poor HRT3 image quality. A total of 75 eyes of 75 patients were included in this study; 39 eyes were glaucomatous and 36 were healthy. All 75 eyes had undergone DRI-OCT wide scan, HRT3 ONH scan, and Humphrey VF testing. Glaucoma subjects were significantly older than healthy subjects with a mean (±SD) age of 64.36 ± 11.59 years and 53.11 ± 15.01 years, respectively (<0.001). The difference in gender and eye between the two study groups was not statistically significant. No difference was found in disc size and imaging quality between the study groups. The demographics and clinical characteristics of each study group are shown in Table 1.

**Table 1:** Demographic characteristics of the study groups. *Continuous variables are expressed as mean ± standard deviation. P-values for comparison between study groups: comparisons were performed using the chi-square test for categorical variables and the Student’s t-test for continuous variables. Statistically significant values with P-value less than 0.05 appear in boldface. HRT3 = Heidelberg Retinal Tomograph 3; OCT = optical coherence tomography; VFI = Visual Field Index; SE = spherical equivalent; D = diopters

	Overall	Healthy (controls)	Glaucoma	P-value
n	75	36	39	
Age (year)*	58.96 ± 14.31	53.11 ± 15.01	64.36 ± 11.59	<0.001
Gender (male/female)	37/38	16/20	21/18	0.416
Eye (right/left)	48/27	24/12	24/15	0.644
Refraction (SE, D)*	-0.50 ± 1.93	-0.80 ± 2.25	-0.21 ± 1.58	0.187
VFI*	89.77 ± 17.28	99.47 ± 0.69	80.82 ± 20.43	<0.001
MD (dB)*	-3.51 ± 5.96	-0.06 ± 1.07	-6.69 ± 6.88	<0.001
HRT3 disc size (mm^2^) *	2.283 ± 0.50	2.23 ± 0.47	2.32 ± 0.52	0.229
OCT topo image quality*	61.95 ± 2.15	61.94 ± 2.04	61.95 ± 2.29	0.497
HRT3 image SD (μm) *	18.23 ± 8.13	17.03 ± 6.43	19.33 ± 9.48	0.226

The best performing HRT3 continuous parameter based on receiver operating characteristic (ROC) curves and AUCs was rim volume (AUC = 0.829, 95% CI = 0.735-0.922), and the best continuous parameter for wide-field DRI-OCT was vertical cdr (AUC = 0.883, 95% CI = 0.805-0.951), followed by total cpRNFL thickness (AUC = 0.862, 95% CI = 0.774-0.951) (Table 2).

**Table 2:** Area under the receiver operating curves and sensitivities and specificities for discriminating between glaucoma and healthy controls (continuous parameters). P comparisons were performed using the method described by DeLong et al. [27]. AUC = area under the receiver operating characteristic curves; CI = confidence interval; cdr = cup/disc ratio; cpRNFL = circumpapillary retinal nerve fiber layer; DRI-OCT = deep range imaging-optical coherence tomography; GPS = glaucoma probability score; HRT3 = Heidelberg Retinal Tomograph 3; Sn = sensitivity; Sp = specificity

		95% CI				
Continuous parameters	AUCs	Lower bound	Upper bound	P-value	Sn at Sp 80%	Sn at Sp 90%
HRT3 parameters						
Rim area (mm^2^)	0.805	0.703	0.907	<0.001	68.4%	63.2%
Rim volume (mm^3^)	0.829	0.735	0.922	<0.001	71.1%	71.1%
Mean RNFL thickness	0.807	0.710	0.903	<0.001	57.9%	44.7%
Linear cdr	0.809	0.708	0.910	<0.001	65.8%	60.5%
Height variation contour (mm)	0.676	0.554	0.798	0.005	50.0%	28.9%
GPS glaucoma probability	0.774	0.668	0.881	<0.001	55.3%	36.8%
DRI-OCT parameters						
Linear cdr	0.849	0.758	0.940	<0.001	73.7%	65.8%
Vertical cdr	0.883	0.805	0.961	<0.001	78.9%	73.7%
Cup volume (mm^3^)	0.740	0.628	0.852	<0.001	60.5%	47.4%
Rim area (mm^2^)	0.857	0.769	0.946	<0.001	76.3%	73.7%
cpRNFL total (μm)	0.862	0.774	0.951	<0.001	78.9%	78.9%
cpRNFL superior (μm)	0.835	0.740	0.930	<0.001	81.6%	68.4%
cpRNFL inferior (μm)	0.841	0.748	0.934	<0.001	73.7%	71.1%

When 80% specificity was evaluated, all wide-field DRI-OCT sensitivities (except the cup volume sensitivity) were higher than the sensitivities of the main stereometric HRT3 parameters (Table 2). The best sensitivities at a fixed specificity of 90% were seen for the wide-field DRI-OCT measurements (cpRNFL total = 78.9%, rim area = 73.7%, vertical cdr = 73.7%, cpRNFL inferior = 71.1%), followed by the HRT3 rim volume measurement (71.1%). The diagnostic sensitivity of DRI-OCT cup volume measurement was moderate both at 80% and 90% specificities (60.5% and 47.4%, respectively).

**Figure 3:**
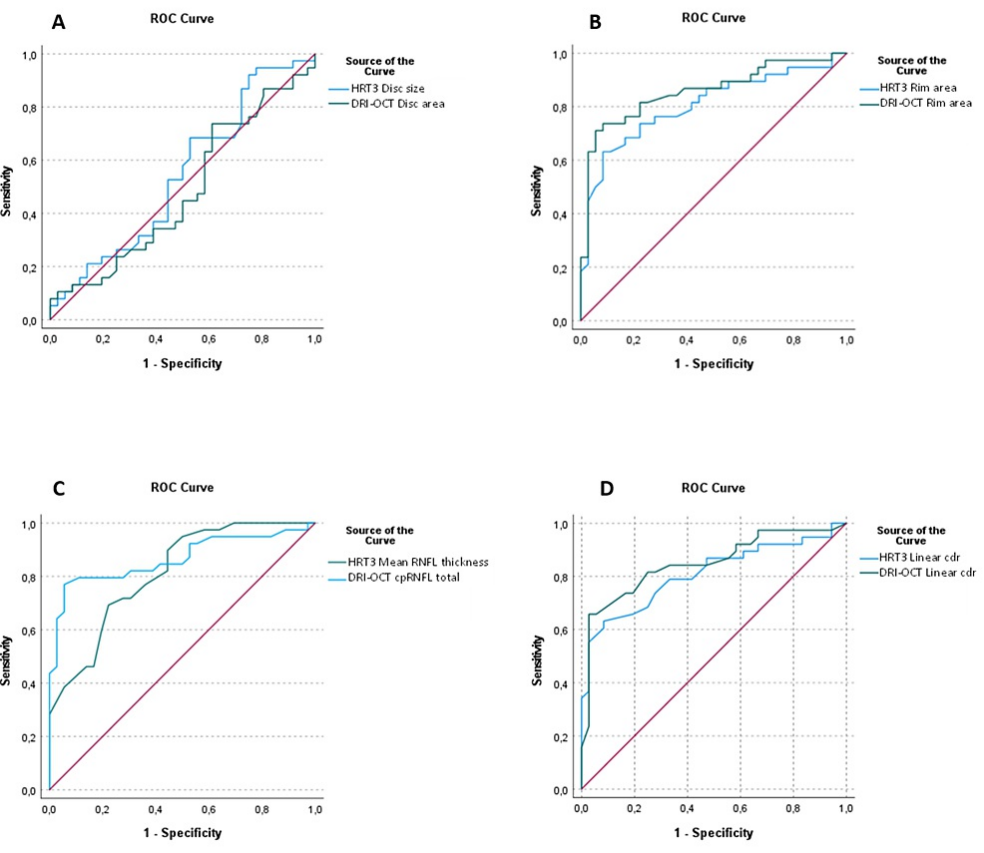
ROC curve for discriminating between healthy and glaucomatous eyes using the HRT3 and SS-OCT (DRI-OCT) for continuous measurements of (A) disc area, (B) rim area, (C) RNFL thickness, and (D) linear cup-to-disc. The blue line represents the SS-OCT (DRI-OCT) measurement and the green line the HRT3 measurement. The AUC values are presented in Table 3. ROC = receiver operating characteristic; HRT3 = Heidelberg Retinal Tomograph 3; SS-OCT = swept-source optical coherence tomography; DRI-OCT = deep range imaging optical coherence tomography; cdr = cup-to-disc; AUC = area under the ROC curves

The AUCs of the continuous parameters that are common to both DRI-OCT and HRT3 (disc area, rim area, linear cdr, RNFL thickness) are shown in Figure 3.

The values and pairwise comparisons of the above-mentioned AUCs are shown in Table 3. No statistically significant difference was seen between the paired measurements.

**Table 3:** Pairwise comparison of AUC (95% CIs) for continuous parameters measured by DRI-OCT and HRT3. The method described by DeLong et al. [27] was used to compare AUCs. *cpRNFL total (DRI-OCT) vs. mean RNFL thickness (HRT3). AUC = area under the receiver operating characteristic curves; CI = confidence interval; cdr = cup/disc ratio, cpRNFL = circumpapillary retinal nerve fiber layer; DRI-OCT = deep range imaging-optical coherence tomography; HRT3 = Heidelberg Retinal Tomograph 3; RNFL = retinal nerve fiber layer

	DRI-OCT	HRT3	P-value
Disc area (mm^2^)	0.482 (0.347-0.617)	0.542 (0.407-0.676)	0.268
Rim area (mm^2^)	0.857 (0.768-0.946)	0.805 (0.702-0.907)	0.340
Linear cdr	0.849 (0.757-0.940)	0.809 (0.708-0.910)	0.273
RNFL thickness (mm)*	0.866 (0.799-0.953)	0.812 (0.717-0.906)	0.306

The sensitivity and specificity of the categorical variables for discriminating between healthy and glaucomatous eyes are presented in Table 4. When the most specific criteria were used, the best performing HRT3 categorical parameter was MRA classification (sensitivity/specificity = 74.4%/94.4%, PPV = 0.93, NPV = 0.79) and the best DRI-OCT categorical parameters were those for the Super-Pixel map (sensitivity/specificity = 82.1%/88.9%, PPV = 0.89, NPV = 0.82) and cpRNFL (12 sectors) (sensitivity/specificity = 79.5%/91.7%, PPV = 0.94, NPV = 0.80) (Table 4).

**Table 4:** Sensitivity and specificity, positive predictive value, and negative predictive value of HRT3 and DRI-OCT categorical parameters. *P-values compared with the Superpixel-200 map (McNemar test). Statistically significant values with P-value less than 0.05 appear in boldface. cdr = cup/disc ratio; cpRNFL = circumpapillary retinal nerve fiber layer; CSM = cup shape measure; DRI-OCT = deep range imaging-optical coherence tomography; GC = ganglion cell; HRT3 = Heidelberg Retinal Tomograph 3; MRA = Moorfields regression analysis; GPS = glaucoma probability score; RNFL = retinal nerve fiber layer; mGCIPL = macular ganglion cell layer and inner plexiform layer; mGCC = macular ganglion cell complex

		Most specific criteria			Least specific criteria		
		Sn/Sp (%)	PPV/NPV	P*	Sn/Sp (%)	PPV/ NPV	P*
HRT3 categorical parameters							
Cup							
	Linear cdr	03/97	0.50/0.48	<0.001	15/97	0.86/0.52	<0.001
	CSM	5.1/100	1.0/0.49	<0.001	23.1/97.2	0.90/0.54	<0.001
Rim							
	Rim area	25.6/100	1.0/0.55	<0.001	43.6/97.2	0.94/0.61	<0.001
	Rim volume	17.9/100	1.0/0.53	<0.001	41.0/100	1.0/0.61	<0.001
RNFL							
	Mean RNFL thickness	2.6/100	1.0/0.49	<0.001	25.6/100	1.0/0.55	<0.001
	Height variation contour	-/100	-/0.48	-	12.8/100	1.0/0.51	-
MRA							
	MRA classification	74.4/94.4	0.93/0.77	<0.001	79.5/80.6	0.82/0.78	0.024
	MRA global	25.6/100	1.0/0.55	<0.001	48.7/97.2	0.95/0.64	<0.001
GPS							
	GPS classification	69.2/75.0	0.75/0.69	0.008	82.1/55.6	0.67/0.74	0.701
	GPS global	53.8/72.2	0.70/0.60	<0.001	79.5/55.6	0.67/0.74	0.458
DRI-OCT categorical parameters							
cpRNFL thickness							
	cpRNFL (4 sectors)	71.8/94.4	0.93/0.76	<0.001	76.9/75.0	0.77/0.75	0.012
	cpRNFL (12 sectors)	79.5/91.7	0.94/0.80	<0.001	87.2/58.3	0.71/0.81	0.549
Macular GC analysis							
	mGCIPL (6 sectors)	61.5/94.4	0.92/0.69	<0.001	69.2/86.1	0.84/0.72	<0.001
	mGCC (6 sectors)	69.2/94.4	0.93/0.74	<0.001	76.9/91.7	0.91/0.79	<0.001
Peripapillary and macular analysis	Superpixel-200 map	82.1/88.9	0.89/0.82		89.7/55.6	0.69/0.83	

When the least specific criteria were used, the two highest sensitivity values among HRT3 categorical parameters were those for the GPS classification (82.1%) and MRA classification (79.5%). Similarly, the two highest sensitivity values among DRI-OCT categorical parameters were observed for the Superpixel-200 map (89.7%) and the cpRNFL (12 sectors) thickness analysis (87.2%) (Table 4).

**Figure 4:**
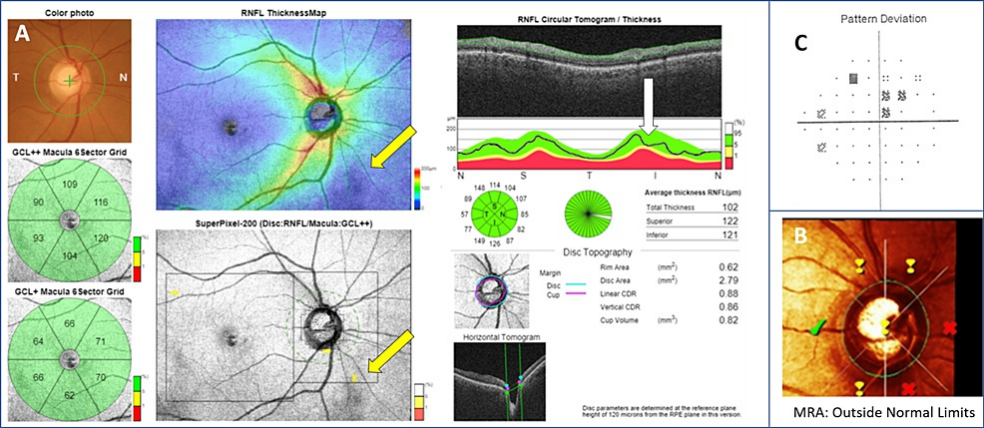
A 65-year-old female with primary open-angle glaucoma in the right eye. Wide-field DRI-OCT green disease (false-negative) in ONH-RNFL assessment. (A) DRI-OCT. Macular ganglion cell analysis classification by DRI-OCT (GCL +, GCL ++) was classified as WNL. The SuperPixel-200 map showed non-specific findings. cpRNFL thickness showed borderline thinning inferiorly that was classified WNL by both the four- and 12-sector analysis. (B) HRT3. In this patient, MRA classification was ONL, and MRA Global was borderline. (C) HFA 24-2 visual field test showing a small superior defect that extends into the central 100 and corresponds to the inferior RNFL depression as well as the inferior MRA sectors classified as ONL. DRI-OCT: deep range imaging optical coherence tomography; ONH = optic nerve head; RNFL = retinal nerve fiber layer; cpRNFL = circumpapillary retinal nerve fiber layer; GCL + = ganglion cell inner plexiform layer (GC-IPL); GCL ++ = GC-IPL plus RNFL; HRT3 = Heidelberg Retinal Tomograph 3; HFA = Humphrey field analyzer; MRA = Moorfields regression analysis; ONL = outside normal limits; WNL = within normal limits

**Figure 5:**
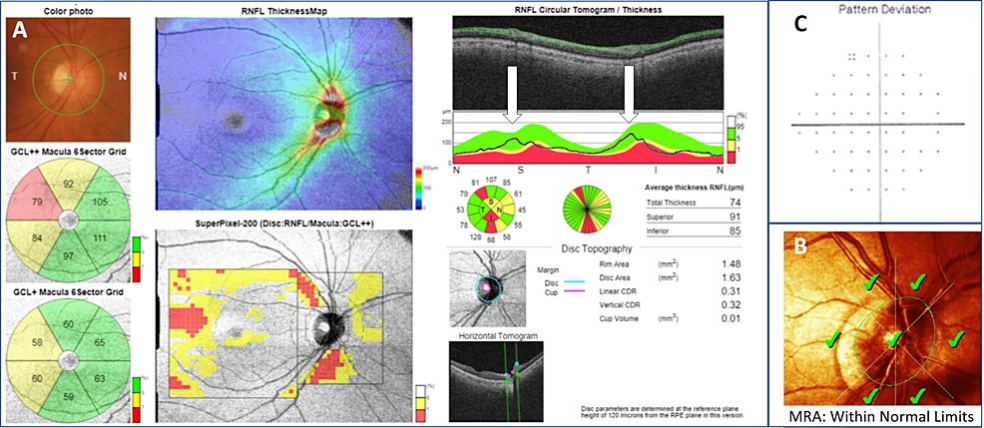
The right eye of a 57-year-old normal female. Wide-field DRI-OCT red disease (false-positive) in ONH-RNFL assessment. (A) DRI-OCT. Macular ganglion cell analysis classification by DRI-OCT (GCL +, GCL ++) was classified as either ONL or borderline. The SuperPixel-200 map showed an area of significant thickness deviation (GC layer + IPL + RNFL) by depicting superior and inferior RNFL defects. cpRNFL thickness showed shifted RNFL peaks (white arrows), while both the four- and 12-sector analysis misinterpreted the shifted bundles as thinning and classified the relevant sectors as ONL. (B) HRT3. In this patient, both MRA classification and MRA global were WNL. (C) HFA 24-2 visual field shows no defects. DRI-OCT: deep range imaging optical coherence tomography; ONH = optic nerve head; RNFL = retinal nerve fiber layer; cpRNFL = circumpapillary retinal nerve fiber layer; GCL + = ganglion cell inner plexiform layer (GC-IPL); GCL ++ = GC-IPL plus RNFL; HRT3 = Heidelberg Retinal Tomograph 3; HFA = Humphrey field analyzer; MRA = Moorfields regression analysis; ONL = outside normal limits; WNL = within normal limits

Our data also showed that DRI-OCT and HRT3 use different methodologies to perform measurements and classify their measurements using different databases, which may also contribute to the manifestation of false-positive and false-negative results. Two examples are provided below for illustrative purposes (Figures 4, 5).

## Discussion

In this study, we evaluated the diagnostic ability of wide-field DRI-OCT thickness measurements (optic disc, RNFL, and macular) to differentiate glaucomatous from healthy eyes and compared them with the six main ONH stereometric parameters as well as with the GPS and MRA classification algorithms of the HRT3. To our knowledge, this is the first report to compare the ability of wide-field DRI-OCT maps with the well-established technology of HRT3 to detect glaucomatous damage. Our results indicate that the diagnostic abilities of the wide-field DRI-OCT maps were better than the six main HRT3 stereometric parameters and comparable to HRT3 automatic classification algorithms (MRA and GPS) for the discrimination of glaucomatous from healthy eyes.

The imaging technologies that we compared are commercially available in clinical practice, and each provide both numerical values and automated glaucoma status classification for the examined ONH, RNFL, and macular parameters. The automated outputs provide useful and attractive characteristics to clinicians who are not glaucoma experts as they minimize the necessity to interpret a large amount of information in the form of images and measurement values and thus facilitate glaucoma diagnosis.

HRT3 is the most recent version of an established imaging diagnostic tool that provides objective and quantitative ONH and RNFL measurements. HRT is a CSLO device for ONH assessment that was first commercially introduced in 1991 and became the standard of care for diagnosing [8,9,28] and monitoring structural changes [10-13,29,30] in glaucoma in the early 2000s. HRT is a well-studied, essential diagnostic imaging technology that was also used as a research tool in two landmark glaucoma studies, namely, the CSLO ancillary study to the Ocular Hypertension Treatment Study (OHTS) [29] and the HRT Ancillary Study to the European Glaucoma Prevention Study (EGPS) [31]. Baseline HRT ONH parameters were found to highly correlate with clinical evaluation and successfully predicted glaucomatous conversion in ocular hypertensive patients [32,33]. A recent meta-analysis reported a median (range) HRT3 sensitivity and specificity for glaucoma diagnosis of 73.6% (30.6-95) and 82.7% (26-100), respectively [34]. The same authors also reported that MRA was the best performing algorithm/parameter of HRT3, with a sensitivity of 0.80 and specificity of 0.85.

Continuous advancements in imaging technology have led to the development of SD-OCT, which has enabled the objective and quantitative assessment of not only ONH but also RNFL and macular GC thickness. Available literature on SD-OCT proved the glaucoma diagnostic abilities of cpRNFL, RNFL thickness deviation map, and macular GC analysis for glaucoma detection [35-37] and reported comparable glaucoma detection abilities between these analyses [38,39]. The more recently introduced SS-OCT has a more rapid scanning speed and uses longer wavelength light sources (generally 1,050 nm) than SD-OCT (840 nm) and thus enables the imaging of deeper ocular layers, namely, the choroid and lamina cribrosa (LC) [15,40-42]. A major advantage of SS-OCT imaging technology is that it allows simultaneous visualization of both the ONH and macula by performing a wide-field single-scan protocol that reduces examination time. The glaucoma diagnostic ability of wide-field SS-OCT (DRI-OCT), cpRNFL, and macular GC (GC - IPL plus RNFL) thickness measurements was successfully shown and their diagnostic accuracy was found to be comparable to that of SD-OCT [17,18]. Additionally, the wide-field SS-OCT (DRI-OCT) SuperPixel RNFL deviation map showed good ability to differentiate between early glaucoma and healthy eyes [43] and performed better than the SD-OCT (Cirrus HD-OCT) RNFL deviation map in diagnosing glaucoma [20].

We believe our study is the first to compare ONH and RNFL measurements from the long-accepted HRT3 and wide-field DRI-OCT maps. We compared these instruments for the glaucoma diagnostic performance of both categorical classification and thickness measurement values. Additionally, the four shared continuous parameters of the two technologies (disc area, rim area, RNFL thickness, and linear cdr) were directly compared (paired measurements).

The three highest values of sensitivity at a similar specificity were achieved by DRI-OCT continuous parameters (vertical cdr, rim area, total cpRNFL) (Table 2). When the continuous parameters that are common to both technologies were assessed, DRI-OCT parameters (except for the disc area) demonstrated better glaucoma diagnostic accuracy (higher AUCs). Interestingly, no statistically significant difference was seen between the paired measurements for the four shared continuous parameters of the two technologies. HRT3 provides only an indirect evaluation of ONH and RNFL thickness based on the arbitrarily software-generated reference plane, which is defined at 50 µm posterior to the average retinal height between 350 and 356 degrees after the contour line is drawn by the operator. Although the indirect assessment of the ONH and RNFL thickness by HRT3 may account for its lower diagnostic accuracy, our results could not detect any significant differences when compared with the measurements obtained from the newer DRI-OCT maps, thus supporting the usefulness of HRT3 ONH assessment in glaucoma management and diagnosis.

Similar to our results, several investigators have compared thickness measurement values between HRT3 and the previous modality of SD-OCT, including Shin et al. [44] who found that Cirrus SD-OCT optic disc parameters had significantly superior glaucoma diagnostic capability than HRT2 parameters. Roberti et al. [45] also reported slightly better diagnostic ability of SD-OCT optic disc parameters comparing HRT3 and RTVue-100. RTVue-100 showed slightly better performance that was statistically significant only for rim volume (p = 0.04). Other investigators showed that SD-OCT (Spectralis) RNFL measurements performed better than HRT3 optic disc topographic parameters in detecting both preperimetric [46] and perimetric glaucomatous damage [47]. Additionally, Cirrus SD-OCT macular GC thickness measurements demonstrated better glaucoma diagnostic ability than HRT3 optic disc parameters and were significantly less sensitive than Cirrus SD-OCT ONH and RNFL measurements [48].

In clinical practice, glaucoma detection by imaging technologies such as DRI-OCT and HRT3 also relies on the normative-based classification and not only on the actual thickness values, therefore providing a useful automated tool that aids glaucoma diagnosis. Using either the most or the least specific criteria, SuperPixel-200 map always showed the highest sensitivity among the categorical parameters of both technologies (82.1% and 89.7%, respectively, i.e., it missed 18% and 10% of eyes with glaucoma, respectively), followed by cpRNFL thickness (12 sectors) (Table 4). The highest sensitivity among HRT3 classification parameters was shown by MRA and GPS classification, thus confirming previous findings [34]. For the least specific criteria, SuperPixel-200 map, cpRNFL thickness, and GPS classification did not perform as well in identifying normal cases (specificity of 55.6%, 58.3%, and 55.6%, respectively) while MRA classification had the higher specificity (80.6%) compared with the other three categorical parameters.

A few studies compared the diagnostic performance of normative-based classification between HRT3 and SD-OCT technology to distinguish normal from glaucomatous eyes. The Glaucoma Automated Tests Evaluation (GATE) study was a large, prospective, comparative diagnostic accuracy study that assessed the diagnostic performance of four imaging tests in identifying glaucoma [49,50]. HRT3-MRA had the highest sensitivity but lower specificity than other imaging tests, while the sensitivity of SD-OCT (Spectralis) was very similar in magnitude to its specificity. Kratz et al. [51] compared the agreement of categorical glaucoma classification between HRT3 and Cirrus SD-OCT. They reported similar glaucoma diagnostic ability of ONH measurements [51] by both technologies and better diagnostic ability of the superior quadrant of RNFL, as measured by Cirrus SD-OCT [52]. They also found excellent to good categorical agreement in ONH parameters and poor agreement in RNFL categorical classification between Cirrus OCT and HRT3. Similarly, Leung et al. [47] showed that SD-OCT (Spectralis) diagnostic classification (global RNFL thickness) had significantly better diagnostic performance than HRT3 (global rim area, vertical cdr, rim-disc area ratio, FSM, and RB discriminant function value).

Our data show that the wide-field maps of DRI-OCT can be useful imaging modalities because they show the RNFL status in a wider area with better diagnostic ability than ONH and RNFL stereometric parameters provided by HRT3. What is becoming apparent, however, is that the two instruments use different methodologies to perform measurements and classify their measurements using different databases, with a definite practical impact on their ability to diagnose pathology. DRI-OCT results interpretation is made easier and quicker by color labeling, which provides the statistical significance of structural loss in comparison to the underlying normative database. Nevertheless, it may also contribute to the manifestation of red-green disease [53,54].

Green classification may be falsely reassuring in selected cases where glaucoma is present, resulting in green disease, as shown in Figure 4. In this case of glaucoma, localized RNFL thinning of the inferior bundle was classified as green by RNFL and macular DRI-OCT maps according to the normative database. Inferior RNFL loss can be observed as a depression of the TSNIT curve (white arrow), while the Superpixel-200 map does not show significant deviations from the normative database thickness values. DRI-OCT green disease could be attributed to the following reasons: (A) This specific inferior RNFL bundle starts from peripheral ganglion cells and its arcuate course is located outside the macular and peripapillary scanned areas (yellow arrows) with available normative thickness data, thus normative comparison is not possible. (B) Because the patient probably had high RNFL thickness values initially (compared to the normative database), the parapapillary bundle section (area with available normative data) with glaucomatous damage was classified as green by both the TSNIT curve and the Superpixel-200 map, despite the RNFL thinning. Interestingly, HRT3 MRA algorithm correctly classified the RNFL thinning which could be attributed to the wider normative database of HRT3 [7,26].

On the other hand, red labeling can result in false-positive results and diagnosis, that is, red disease. Figure 5 illustrates a case of DRI-OCT false-positive glaucoma diagnosis caused by displaced RNFL bundle peaks. There are two peaks in the cpRNFL thickness profile (TSNIT curve) that correspond to the thicker RNFL bundles, namely, the supratemporal and infratemporal peaks. Temporally or nasally shifted RNFL peak thickness profiles represent well-recognized normal anatomic variations of RNFL thickness distribution [55,56]. It is also well documented that the mean angle between the fovea- Bruch’s membrane opening (BMO) center (FoBMO) axis and the horizontal axis of the image frame can vary from 60 to -170 among individuals [57]. Therefore, the current OCT method of assigning sectors relative to the fixed horizontal and vertical axes of the imaging device could result in anatomical misalignment of ONH, RNFL, and macular imaging data, as well as incorrect normative comparison, thus causing red disease artifacts in healthy individuals [58]. To compensate for this, Chauhan and Burgoyne [59] proposed that both image acquisition and analysis should be performed according to the specific FoBMO axis of an individual to ensure the correct anatomical correspondence of all ONH, RNFL, and macular thickness measurements. Kim et al. [60] confirmed the clinical value of FoBMO axis by applying a new wide-field normative database considering the Fovea-Disc relationship that significantly improved the diagnostic performance of DRI-OCT in Korean patients.

This study had several limitations. First, the sample size was relatively small. This did not allow a meaningful subanalysis for the effect of possible confounding factors (i.e., age, gender, axial length, glaucoma severity) on the diagnostic performance of wide-field DRI-OCT protocol. More data from larger studies are needed to assess the usefulness of wide-field protocol in real-world clinical settings. Second, the normal group was younger than the glaucoma group. Given the fact that RNFL thickness significantly decreases with age [61,62], this could influence the sensitivity of our study. Lastly, the inclusion in our case-control study was based on normal subjects and definite glaucoma cases and so the outcomes may be biased and not represent the whole spectrum of glaucoma presentation (i.e., glaucoma suspects). Therefore, the performance of wide-field DRI-OCT maps could be overestimated due to spectrum bias [63].

As noted above, this is the first study to compare the diagnostic performance of wide-field DRI-OCT maps and HRT3 ONH measurements for the same population. Our results suggest that Superpixel-200 map of DRI-OCT showed better glaucoma diagnostic performance than HRT3 automated diagnostic classification. Therefore, shows the advantage of DRI-OCT wide-area scan and stresses the importance of gathering thickness data beyond the peripapillary area. This study also shows that, while different imaging modalities become more readily available, ONH analysis with HRT3 (MRA, GPS) still provides valuable diagnostic information that also allows for long-term monitoring of glaucoma.

## Conclusions

Our results suggest that wide-field DRI-OCT automatic classification is a fast and effective diagnostic tool that is easy to read and interpret. However, relying solely on wide-field DRI-OCT imaging (as used in this study) as a diagnostic test is not recommended as some patients may be misclassified. Clinicians should be familiar with the limitations of the wide-field DRI-OCT protocols and possible scanning artifacts. We encourage clinicians rather implement a multimodal imaging approach that includes the anatomical examination of both ONH (HRT3) and RNFL/GC (DRI-OCT wide-field) in combination with functional and clinical evaluation of ONH and RNFL to improve glaucoma diagnosis and management. Future studies are needed to further assess the clinical value of wide-field DRI-OCT imaging modality and its role in diagnosing, monitoring, and treating glaucoma.
